# Prostaglandin E_2_ and PD-1 mediated inhibition of antitumor CTL responses in the human tumor microenvironment

**DOI:** 10.18632/oncotarget.21155

**Published:** 2017-09-22

**Authors:** Jie Miao, Xu Lu, Yuefeng Hu, Chunmei Piao, Xuan Wu, Xuesong Liu, Caiting Huang, Yue Wang, Dan Li, Jingwei Liu

**Affiliations:** ^1^ Department of Radiology, Beijing Obstetrics and Gynecology Hospital, Capital Medical University, Beijing, China; ^2^ Department of Oncology, Beijing Biohealthcare Biotechnology Co.,Ltd, Bejing, China; ^3^ Department of Interventional Therapy Center, Beijing Shunyi Distinct Hospital, Bejing, China; ^4^ Department of Oncology, Beijing Anzhen Hospital Affiliated to the Capital Medical University, Beijing Institute of Heart Lung and Blood Vessel Diseases, Bejing, China

**Keywords:** cytotoxic T lymphocytes (CTL), PGE2, PD-1, tumor microenvironment

## Abstract

Accumulating evidence indicates that inflammation plays a critical role in cancer development; however, mechanisms of immunosuppression hinder productive anti-tumor immunity to limit immunopathology. Tumor-specific cytotoxic T lymphocyte (CTL) dysfunction or exhaustion by upregulating inhibitory receptors such as programmed cell death 1 (PD-1) in tumor-bearing hosts is one such mechanism. Identification and blockade of the pathways that induce CTL dysfunction has been shown to partially restore CTL function in tumor-bearing hosts. Cyclooxygenase-2 (COX-2) is a rate-limiting enzyme for prostanoid biosynthesis, including prostaglandin E_2_ (PGE_2_), and plays a key role in both inflammation and cancer. The disruption of COX2/PGE2 signaling using COX_2_ inhibitors or PGE2 receptors EP2 and EP4 antagonists, combined with anti-PD-1 blockade was therapeutic in terms of improving eradication of tumors and augmenting the numbers of functional tumor-specific CTLs. Thus, COX2/PGE2 axis inhibition is a promising adjunct therapy to PD-1 blockade for immune-based therapies in cancer.

## INTRODUCTION

Although tumor-specific cytotoxic T lyphocytes (CTLs) is considered to manifest the host immunologic response to tumor antigens inside the tumor tissue micro-environment [1], another process that is equally indispensable is host-driven immune depression, which attenuates anti-tumor immunological reactions [2]. Certain immunosuppressive mechanisms of tumor-bearing hosts may be reflected in the host-driven immune depression. Identifying factors that govern the functions of tumor-specific CTLS are therefore necessary to effectively prevent cancer recurrence.

As shown in infectious disease models, continuous challenge of chronic antigen often result in ineffective tumor-specific CTLs responses due to functional exhaustion and may lose their ability to produce key cytokines that play a pivotal role in maintenance of CTLs memory [3, 4]. A few negative CTL regulators including regulatory T cells (Tregs), TGF-β, and IL-10 and the potential inhibitory receptors PD-1, 2B4, CTLA-4, PirB, Tim-3 and CD160 have been identified in studies that performed whole genome expression profiling [5, 6]. A considerable amount of evidence has recently revealed that the expression of these receptors plays an important role in modulating multiple functional aspects of the exhaustion of CTLs [7, 8]. Also, this expression may result in physical reduction of certain tumor-specific CTL populations [9]. Therefore, the blockade of several repressive pathways, such as TGF-β, PD-1, or IL-10, can enhance the number of CTLs and efficacious functions to protect against cancer and chronic infections [6, 10–14]. It will be important to identify the factors that govern CTL exhaustion to develop more efficient treatments.

Our study showed that tumor-derived prostaglandin E_2_ (PGE_2_) plays an important role in depressing CTL function and survival in patients receiving cancer immunotherapy. PGE_2_ is a potent lipid mediator that is synthesized from arachidonic acid using sequential actions of cyclooxygenases (COX; constitutively active cyclooxygenase COX-1 and inducible COX2) and PGE synthases (PGESs), which plays an important role in both inflammation and tumorigenesis [15, 16]. PGE_2_ and its key synthesizing enzyme COX2, which are often overexpressed in stomach, pancreatic, lung, breast, and colorectal cancers [17, 18], have been implicated in this regard given the ability of COX2 blockade to enhance the effectiveness of cancer vaccination [19, 20]. Notably, PGE_2_ has also been shown to suppress CTLs survival, type I interferon production and cytotoxicity *in vitro* [21], and treatment of tumor-bearing mice with COX-2 inhibitors and PD-1 monoclonal antibody (mAb) improve antitumor immunity [22]. The biological actions of PGE_2_ are mediated via 4G-protein-coupled receptors (EP1-EP4), of which EP2 and EP4 have also been shown to be involved in the elevation of exhausted CTLs [23, 24], and it will be essential to detect the possibility of the suppression of PGE_2_ signaling. In this study, we identified that targeting the PGE_2_ signaling pathway constitutes a useful additions to PD-1 blockade, to enhance the effectiveness of cancer immunotherapies.

## RESULTS

### COX-dependent prostanoids account for the immunosuppressive effects

**Figure 1:**
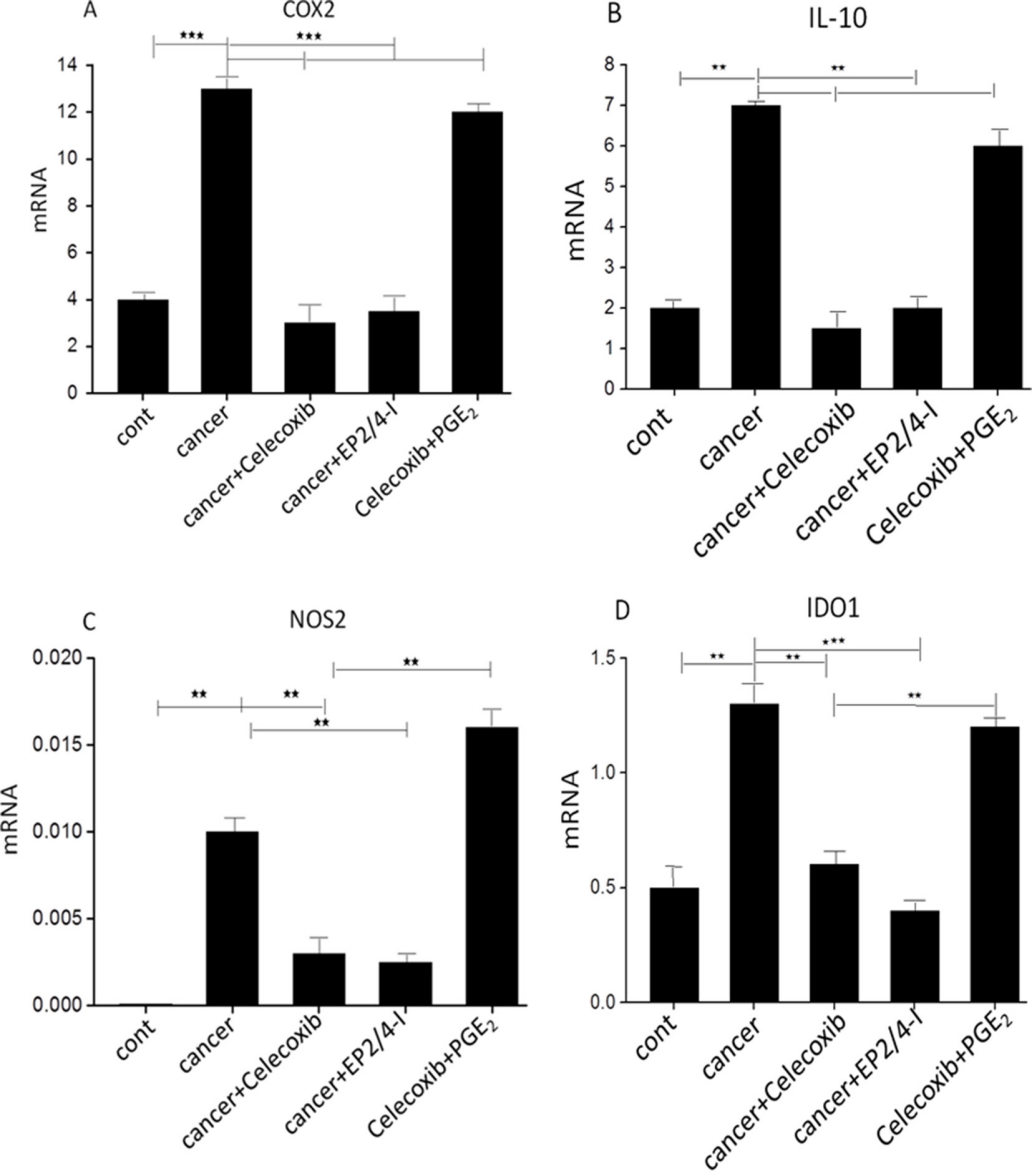
Induction of immunosuppressive factors by cancer-associatedascites cells (**A–D**) Expression of immunosuppressive factors in cancer-associated ascites cells pretreated (24 hours) or not with celecoxib, the EP2/EP4 antagonist. The addition of synthetic PGE_2_ to celecoxib-pretreated ascites cells isolated from cancer patients restores immunosuppressive functions. Neither celecoxib nor the EP antagonists showed any cytotoxic effects at the concentrations used. All data (panels A-D) were confirmed in 3 independent experiments and a single representative experiment with different donors as mean ± SD. ^⋆^*P* < 0.01; ^⋆⋆^*P* < 0.001.

Given the known pleiotropic suppressive effects of the COX2/PGE_2_ axis on tumor-specific CTLs immunity, and the documented ability of blockade of tumor-associated COX2 to skew toward a type-1 cytokine response [25, 26], we anticipated a negative correlation between COX2 and the local development CTL cells associated with the cancer microenvironment. Several lines of evidence have shown that inhibition of the differentiation of monocytes into functional CD1a^+^ DCs is associated with the induction of endogenous COX2-derived prostanoids [27, 28]. The addition of synthetic PGE_2_ is sufficient to redirect the differentiation of functional DCs toward monocytic myeloid-derived suppressor cells (MDSCs) phenotype and CTL-suppressive function [27]. We investigated the involvement of PGE_2_ in the initial primary of naïve CD8^+^ T cells (Supplementary Figure 2) and development of tumor-derived CTLs associated with the cancer microenvironment. As shown in Figure 1, the frequencies of tumor-associated immunosuppressive factors, including the COX2, IL-10, NOS2 and IDO1 mRNA was abrogated by the addition of COX2 inhibitor during the generation of CM from cancer ascites cells. In accordance with the critical requirement for PGE_2_ in the ability of the CM from cancer ascites cells to induce these suppressive factors implicated in tumor-associated immune dysfunction, the inhibition of COX2 abrogated the ability of CM from cancer ascites cells to induce COX2-PGE_2_ feedback.

**Figure 2:**
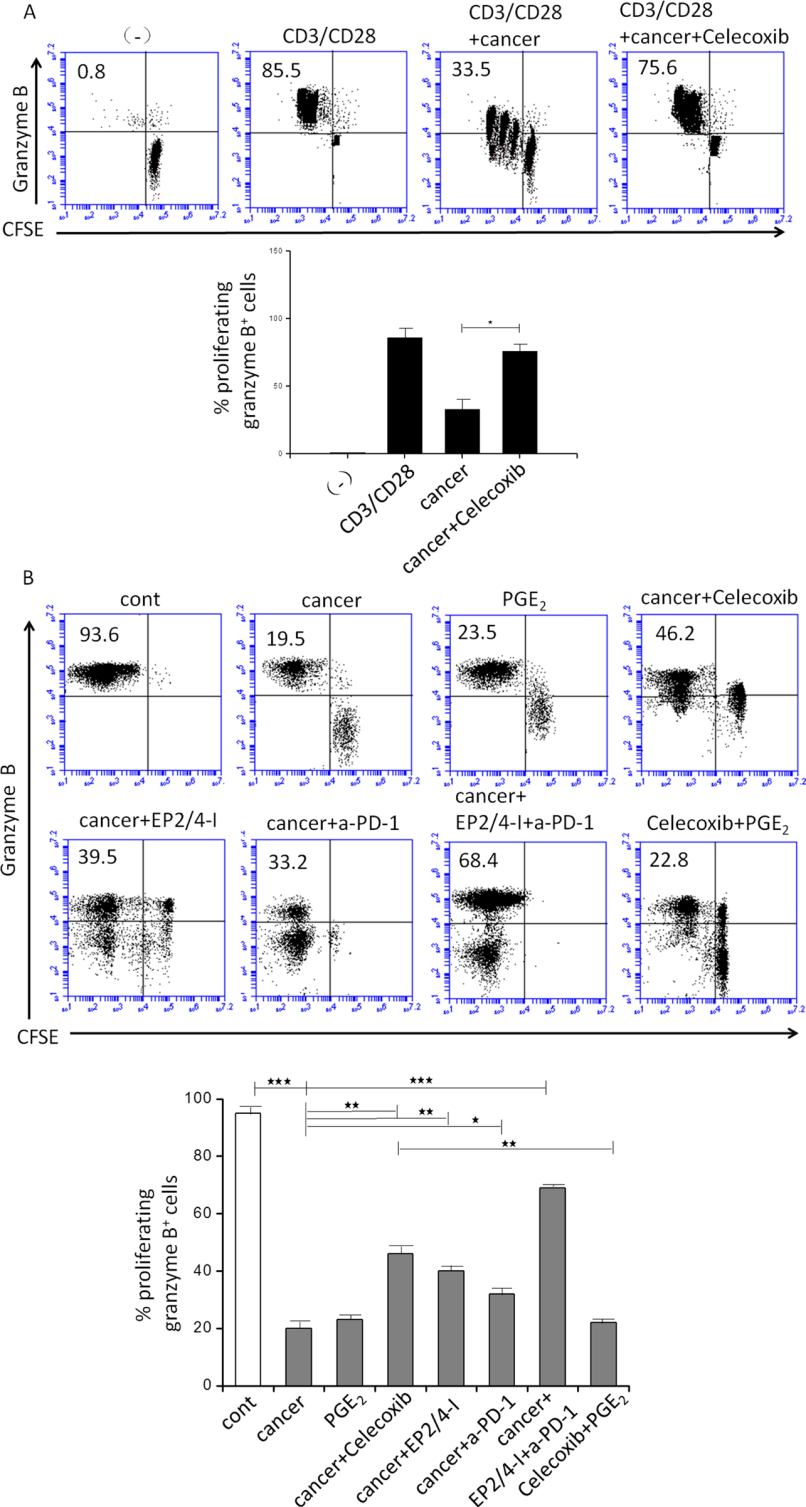
Effect of inhibition of COX2, the EP2 and EP4 (EP2/4-I), and PD-1 on CD3/CD28-induced proliferation of granzyme B^+^ CTL (percentages) from autologous naïve CD8^+^ T cells (**A**) Immmunosuppressive effects of cancer-associated ascites cells on naïve CFSE-labeled CD8^+^ T cells primed by CD3/CD28 and stained for granzyme B (marker of CTL status). Celecoxib pretreatments of ascites cells abolishes their suppressive impact on CD3/CD28-activated naïve CD8^+^ T cells. Percentages indicate the fraction of proliferating granzyme B^+^ CTL cells. (**B**) Cancer-infiltrating primary cell condition medium (CM; 1:1 ratio respectively) was generated in the presence or absence of celecoxib, the EP2/EP4 antagonist (EP2/4-I), and anti-PD-1 (a-PD-1). Inhibition of COX2, the EP2 and EP4 and PD-1 counteracts the suppressive impact of ascites cells on CD3/CD28-activated naïve CD8^+^ T cells (*n* = 3). All data were confirmed in at least 3 independent experiments. Histograms present data from a single representative experiment with different donors as mean ± SD. ^⋆^*P* < 0.05; ^⋆^*P* < 0.01; ^⋆⋆^*P* < 0.001.

**Figure 3:**
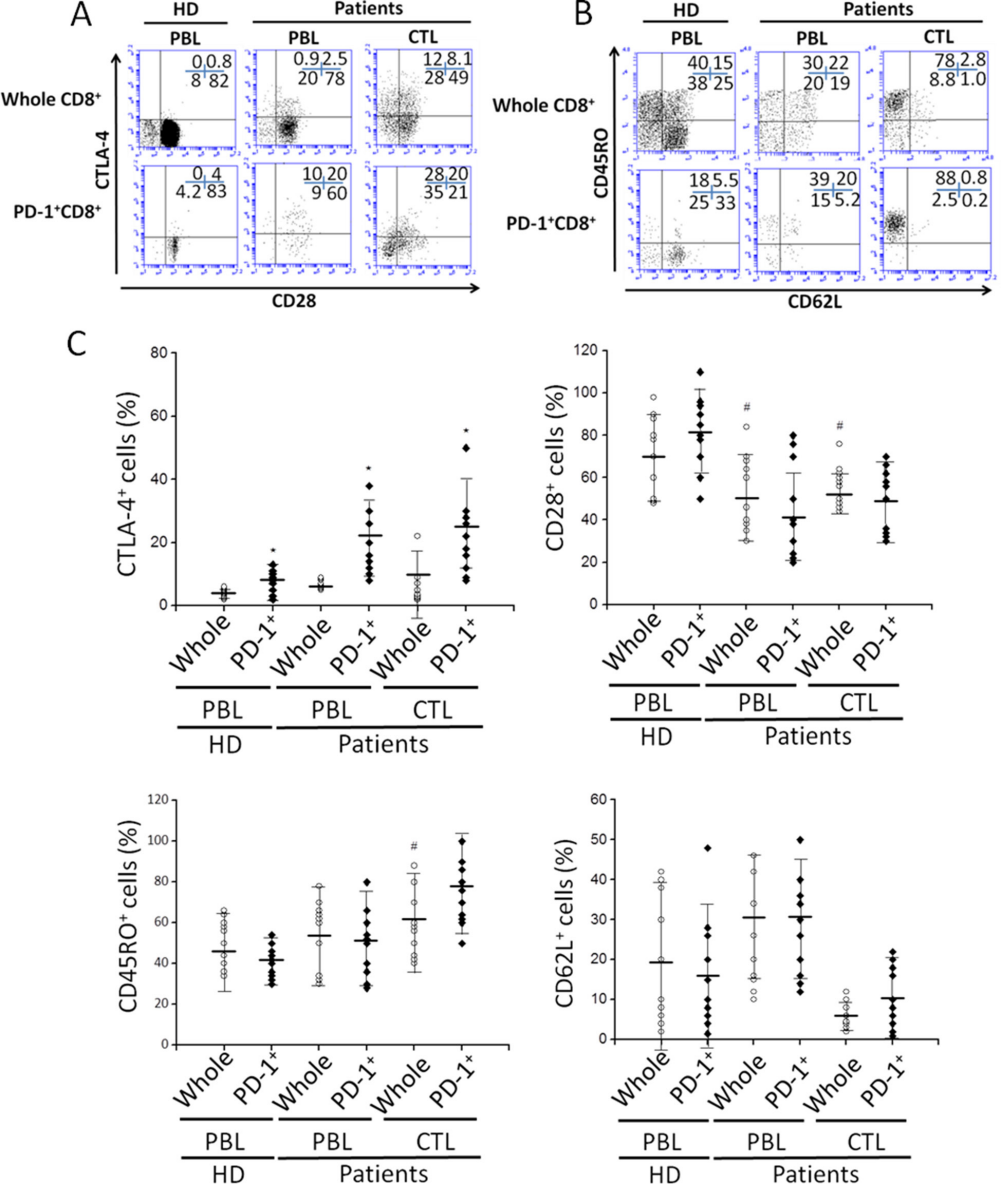
The phenotypic characterization of PD-1^+^CD8^+^ cells in PBLs and tumor infiltrating CTLs (**A** and **B**) The surface expression of CTLA-4, CD28, CD45RO, and CD62L on whole CD8^+^ or PD-1^+^CD8^+^ cells. Numbers indicate the percentage of cells in each quadrant. (**C**) The expression of indicated molecules from all specimens of healthy donor or cancer patients. ^#^*P* < 0.05 compared with whole CD8^+^ cells of healthy donors’ PBLs. ^⋆^*P* < 0.05 compared with whole CD8^+^ cells of each corresponding tissue.

Consistent with these observations, we further demonstrate that celecoxib treatment also reversed the ability of CM from cancer ascites cells to suppress the CTLs proliferation and their acquisition of granzyme B-containing cytolytic granules (Figure 2A). The previous evidence has shown that exhausted CTLs increased expression of a few inhibitory receptors including the PGE_2_ receptors EP4 and EP2 using whole genome expression profiling [24]. Indeed, EP2 and EP4 are up-regulated on CTLs cultured with CM from cancer ascites cells (Supplementary Figure 3). We further observed that the EP2/4 antagonist, generated results similar to celecoxib, indicating that binding of PGE_2_ to these 2 receptors of the CTLs is involved in its suppressive functions (Figure 2B). To further analyze tumor infiltrating CTLs, we analyzed these cells by staining with cell-surface molecules that are related to T-cell responsiveness. Aside CTLA-4 expression, there were no clear differences in the expression of CD28, CD45RO, and CD62L between PD-1^+^ cells in tumor infiltrating CTLs at the tumor site and whole CD8^+^ T cells (Figure 3). *Ex vivo*, we also assessed the effective role of antigen-nonspecific CD8 function in PBLs and tumor infiltrating CTLs. Even though the induction of IFN-γ was nearly the same among the samples, IL-2 and TNF-α production from CTLs was substantially lower than from homologous PBLs. This indicated that there was damaged effective function of CD8^+^ T cells at the tumor site. Also, tumor infiltrating CTLs implicated considerably more PD-1^+^CD8^+^ T cells than PBLs that originated from patients or healthy individuals (Supplementary Figure 4A and 4B).

### Dual blockade of PGE_2_ and PD-1 pathway renovates tumor-specific CTLs responses

**Figure 4:**
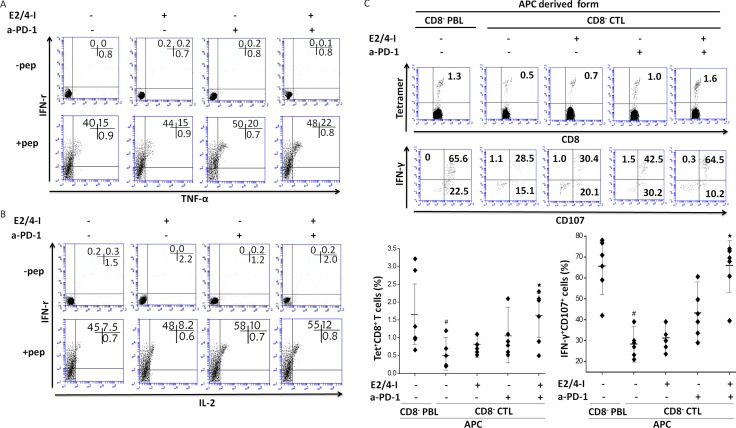
Dual blockade of EP2/4 and PD-1 pathways increases the frequency and enhances effector function of tumor antigen-specific CTLs (**A** and **B**) Whole CTLs derived from cancer patients were cultured in the presence or absence of indicated blocking antibodies. After 2 days of culture, peptide-specific cytokine productions (IFN-γ/TNF-α or IFN-γ/IL-2) from NY-ESO-1^+^ tetramer^+^ cells were analyzed. (**C**) The frequency of tetramer^+^CD8^+^ T cells and peptide-specific IFN-γ/CD107 expression *in vitro* presensitization with or without EP2/4 and PD-1 blockade. ^#^*P* < 0.05 compared with cells stimulated with PBL-derived APCs. ^⋆^*P* < 0.05 compared with untreated CTLs or CTLs treated with a single antagonist. The result shown is representative of three independent experiments from two patients.

Our observations, along with the previous studies that targeting two or more inhibitory pathways can strengthen tumor-specific CTLs responses in cancer [29, 30], presented the possibility that simultaneous blockade of PGE_2_ and PD-1 signaling pathways may verify the most effective way to rescue the immunodominant CTLs from exhaustion in the presence of chronic antigen exposure. For this reason, we investigated the effection of combined blockade of PGE_2_ and PD-1 in tumor infiltrating CTLs, which were isolated from epithelial OvCa tissues. Whole tumor infiltrating CTLs were cultured with blocking antibodies to the PD-1 pathway or PGE_2_ for 48 h. PGE_2_ pathways blockade were composed of the combined of EP2 and EP4 antagonist. We next examined the capability of HLA-A2/NY-ESO-1_157-165_-specific CTLs to produce antigen specific pro-inflammatory cytokines (IFN-γ, IL-2, TNF-α) with intracellular staining. Figure 3A shows tumor-specific IFN-γ-producing CTLs were observed in the presence of NY-ESO-1_157-165_ peptides. A small proportion of the cytokines were IFN-γ/TNF-α^+^ or IFN-γ/IL-2^+^ (Figure 4A and 4B).

We next determined whether simultaneous blockade of PD-1 pathways and PGE_2_ in the presence of antigens could increase NY-ESO-1-specific CTLs responses that were primed by tumor-derived APCs (Supplementary Figure 5). We first measured *ex vivo* frequencies of cytokine-producing CTLs and NY-ESO-1-specific tetramer^+^ CTLs are shown in Figure 4C. We found significant reductions in the frequencies of cytokine-producing CTLs and NY-ESO-1-specific tetramer^+^ CTLs compared to those primed by PBL-derived APCs. There were no significant differences, however, in effector functions between PGE_2_ or PD-1 blockade alone and those that were untreated. Of note, the frequencies of NY-ESO-1^+^tetramer^+^ cells and cytotoxic markers (CD107 and IFN-γ )-expressing CTLs with combined blockade of PD-1 pathways and PGE_2_ were significantly higher than CTLs with a single antagonist or untreated cells (Figure 4C). In summary, our findings showed that dual blockade of PD-1 signaling and PGE_2_ during priming of tumor-specific CTLs with tumor-derived APCs effectively recovered CTLs responses to levels that were detected with peripheral blood derived APCs.

## DISCUSSION

Considerable evidence that has been accumulated over the last two decades has shown that neoplastic development is associated with an immunoediting process. However, cancer immunoediting can lead to the selection of tumors that engender immunosuppressive factors, which depress the initial type I IFN-dependent innate immune cell activation and/or inhibit the following CTL activity against tumor antigens. Prostanoids were observed to be implicated in tumorgenesis through the promotion of angiogenesis, tumor cell survival, and invasion. As such, tumor cell derived prostanoid production enhances immune evasion. Also, pharmacologic inhibition of COX2 was recently reported to reduce the risk of several cancers. This might help to increase anti-cancer immunity and provide useful additions to immune-based cancer therapies, especially those based on immune checkpoint blockade. In this study, we demonstrate that COX2/PGE_2_ axis impairs CTL effector functions and implicate this pathway could cause CTLs exhaustion in cancer. Blockade of COX2/PGE_2_ signaling through celecoxib treatment reversed the ability of ascites cells to suppress the induction of CTLs. Furthermore, we showed that combined inhibition of PD-1 signaling pathways and COX2/PGE_2_ had additive effects in enhancing CTL function and numbers, partly by recovering the immunodominant populations of tumor-specific CTLs and those are typically deleted. Thus, our findings support simultaneously targeting of COX2/PGE_2_ and PD-1 signaling as an efficacious treatment for cancer where CTLs exhaustion in human tumor microenvironment (TME) is known to occur.

Many suppressive pathways, including the COX2, IL-10, NOS2 and IDO1 mechanisms described here, have been implicated in tumor-associated immune dysfunction within the human TME. In this study, we identified PGE_2_ as a primary tumor-associated inflammatory mediator responsible for the tumor-induced up-regulation of endogenous COX2 within the human TME. Overproduction of PGE_2_ by cancer cells, stroma, and infiltrating myeloid cells, primes naïve human T cells for enhanced production of anti-inflammatory cytokines and increased inhibition of proinflammatory cytokine levels through COX2. Until recently, a few studies directly indicate that blocking COX2/PGE_2_ signaling has a profound effect on dysfunctional CTLs in patients going through immunotherapy for viral infection or cancer, indirect evidence suggests that therapies through inhibition of COX-2/PGE_2_ signaling may enhance CTLs function.

It is known that reversal of tumor-specific CTLs dysfunction is a novel and promising for inducing eradiation tumors. Involvement of the PD-1 pathway has been shown to be a primary marker for exhausted T cells. It was shown recently that a PD-1/PD-L1 pathway blockade increased the frequencies of tumor antigen-specific cytokine producing CTLs [31, 32]. In our study, we assessed a coinhibitory pathway blockade under unique stimulating conditions *ex vivo* and we analyzed the differentiation states of CTLs to observe different outcomes. Whereas a PGE2 pathway blockade promoted the frequency of polyfunctional tumor and antigen-specific CTLs primed by APCs, the effects were observed using a PD-1 signaling blockade stimulated with or without APCs. The high expression of PD-L1 (the ligand for PD-1) on tumors cells is strongly associated with poor prognosis and therefore these findings would be considerable *in vivo* significance [33].

Of note, we found that a combined blockade of PD-1 and PGE_2_ pathways enhanced the frequency of polyfunctional and tumor antigen-specific CTLs. These effects were most significant under stimulating conditions in the company of tumor derived APCs. This was less so when antigen stimulation was absent. Peripheral blood and tumor-derived CTLs that were cocultured with tumor-derived APCs led to generate the CTLs with diminished effector function, it appears that the hyporesponsive phenotype was determined with infiltration of the tumor-specific T cells into the tumor tissue.

In conclusion, considering that the combined blockade of PGE_2_ and PD-1 pathways resulted in substantially reversal of CTLs effector function, our studies indicate that therapeutic strategies for enhancing antitumor CTL function could be accomplished by targeting inhibitory pathways as treatment for some chronic diseases.

## MATERIALS AND METHODS

### Media and reagents

Serum-free AIM-V medium (Invitrogen) was used to generate DCs and Iscove's Modified Dulbecco's Medium (IMDM; Invitrogen) with 5% human AB serum (Gemini) was used for CTL production experiments. The PGE_2_ synthesis inhibitors celecoxib (BioVision) were used at concentrations of 20 μM. The concentrations used did not affect viability in cell cultures, as confirmed by live cell counts. PGE_2_ were obtained from Sigma-Aldrich and used at 10^-6^M. AH6809 (EP2/1 antagonist: EP2 antagonist known to be also a weak inhibitor of EP1), AH23848 (EP4 antagonist) were all purchased from Sigma-Aldrich and used at a 20 μM concentration. The concentrations used did not have any significant impact on the viability of cultured cells, as determined by the live cell counts.

### Cell culture

Tissue specimens, ascites fluids, and peripheral bloods were obtained from patients undergoing surgery for twelve patients with advanced epithelial ovarian cancer (OvCa) in stage III or IV and peripheral bloods from normal volunteers in accordance with a protocol approved by the institutional review board. All patients had given written consent. PBLs from healthy donors and PBLs, tissue specimens and/or ascites fluids used in this study were obtained from patients with no prior immunotherapy.

Naïve CD8^+^CD45RA^+^CD45RO^-^ T cells were isolated from PBMCs by negative selection using the naïve CD8^+^ T-cell enrichment cocktail (Stem Cell Technologies). CD8^+^ T-cells were stimulated with CD3/CD28 Dynabeads (5ul/ml, Invitrogen) in the presence or absence of cancer-infiltrating primary cell conditioned medium (CM). CFSE staining of CD8^+^ T cells (Invitrogen) was performed according to the manufacturer's instruction. On days 4–5, expanded CD8^+^ T cells were analyzed for the expression of granzyme B expression and proliferation.

### Blocking antibody treatment

Tumor infiltrating CTLs derived from cancer patients were cultured in complete medium in the presence or absence of 20μM the EP2/EP4 antagonist (EP2/4-I), 10 μg/ml anti-PD-1 (a-PD-1, J116) mAbs (eBioscience) [34], or a combination of these mAbs. After 2 days of culture, multicytokine production from tetramer^+^ cells was determined by intracellular cytokine staining. Isolated CD8^+^ T cells (5 × 10^5^) were cocultured with NY-ESO-1 peptide-pulsed CD8^-^CD4^-^cells (APCs, 5 × 10^5^) derived from PBLs or CTLs, and blocking antibodies were added to the culture at days 0 and 4. Eleven to fifteen days after culture, the frequency of tetramer^+^ cells and the effector function against peptide were determined by intracellular cytokine staining.

### *Ex vivo* staining

Cells isolated from PBLs and tumor infiltrating CTLs were stained with mAbs against CD8, CTLA-4, CD28, CD45RO, and CD62L (BD Biosciences), and analyzed by FACSCanto II (BD Biosciences).

### *Ex vivo* cell analysis of NY-ESO-1-specific CD8^+^ cells

Phycoerythrin- or allo-phycocyanin-cojugated HLA-A*0201/NY-ESO-1_157-165_ tetramers were prepared as previously described [35]. Cells were stained with tetramers and mAbs against CD8 (BD Bioscience), PD-1 (eBioscience). Tetramers were provided by the department of Agricultural Science Research Institute for cancer research. Cytokine production from tetramer^+^ cells or tumor infiltrating CTLs was determined as described previously [36]. NY-ESO-1-specific CD8^+^ T cells in PBLs were determined as described previously [36].

### ELISA

The cytokine production from CD8^+^ cells in PBLs or tumor infiltrating CTLs of patients were measured in the serum by ELISA analysis, as described previously [36].

### Gene expression by qRT-PCR

For qRT-PCR, mRNA was isolated from ~500,000 sorted cells following the unstruction following the instructions provided with the Qiashredder and RNeasy Kits (QIAGEN). cDNA was then synthesized using SSRTII (Invitrogen). TaqMan analysis was performed on the StepOnePlus system (Applied Biosystems). The expression of each gene was normalized to HPRT1 and is expressed as the fold increase (2^-ΔCT^), where ΔCT = CT_(Target gene)_-CT_(HPRT1)_.

### Statistical analysis

All data was evaluated using Prism Version 5 software (GraphPad) . Comparison between paired and unpaired groups was performed using the appropriate Student's *t* test. A linear correlation between 2 continuous variables was tested with the R^2^ coefficient of determination. When indicated, the data from multiple different patients and control donors are expressed as meas and SD from n donors, and were confirmed in multiple independent experiments, described in the figure legends. *P* < 0.05 was defined as statistically significant.

## SUPPLEMENTARY MATERIALS FIGURES


