# Supplementary material for: Prostaglandin E2 and PD-1 mediated inhibition of antitumor CTL responses in the human tumor microenvironment
Source: Oncotarget. 2017 Sep 22;8(52):89802–10. doi: 10.18632/oncotarget.21155 (PMC5685710; doi:10.18632/oncotarget.21155)
Supplement: Supplementary file 1 [file oncotarget-08-89802-s001.pdf]

Prostaglandin E_2 and PD-1 mediated inhibition of antitumor CTL responses in the human tumor microenvironment

SUPPLEMENTARY MATERIALS

1) To determine antigen-nonspecific CD8 function in PBLs and tumor infiltrating CTLs. The production of IFN-γ, TNF-α, and IL-2 by CD8+ T cells from these specimens was compared after stimulation with anti-CD3/anti-CD28 mAbs. As shown in Supplementary Figure 1, IFN-γproduction was almost similar among the samples. However, we found that TNF-αand IL-2 from CTLs were significantly lower than from autologous PBLs.

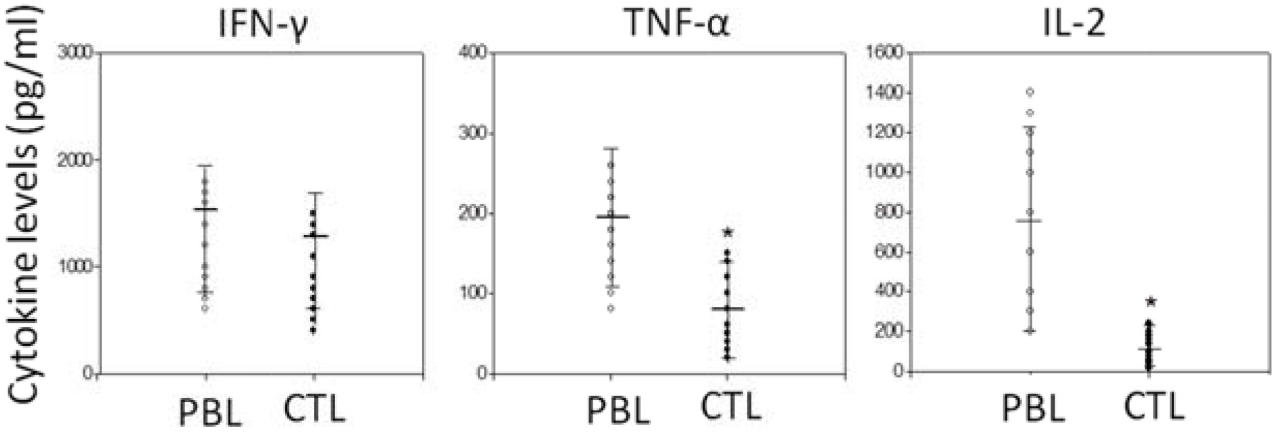

Supplementary Figure 1: Cytokine production from CD8+ cells in PBLs or tumor infiltrating CTLs of six patients. $CD8^+$ cells were isolated from specimens and stimulated with anti-CD3/anti-CD28 mAb-coated beads for 24 h and the cytokine levels in the supernatant were measured by ELISA. *$P < 0.05$ compared with PBLs.

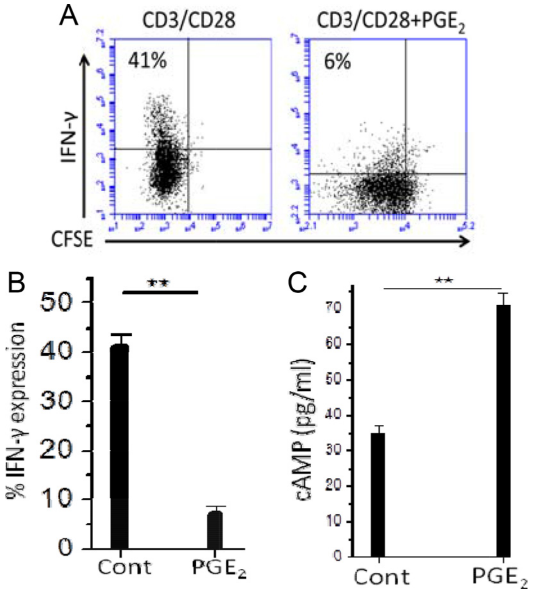

Supplementary Figure 2: PGE$_2$ directly suppresses effector functions of CD3/CD28-induced CTLs in a cAMP-dependent manner. The direct effect of PGE2 on CTLs priming was examined by culturing CFSE-labeled $CD8^+$ T cells in the presence of immobilized anti-CD3 and anti-CD28 mAb with or without 10^{-6} M PGE2 (**A** and **B**). Although untreated CD8+ T cells proliferated and elaborated IFN-γ, the presence of PGE2 resulted in a reduction in proliferation, with many more undivided CFSE-high cells, and a significant decrease in IFN-γ expression. To establish whether or not PGE2 suppresses naïve $CD8^+$ T cells primed by CD3/CD28 in a cAMP-dependent manner, cells were lysed, and intracellular cAMP concentrations were measured using an ELISA. As shown in (**C**), following PGE2 treatment, cAMP expression amongst cells rose by around two fold compared to untreated cells. Statistical analysis was performed by unpaired two-tailed *t*-test. $^{**}P < 0.01$.

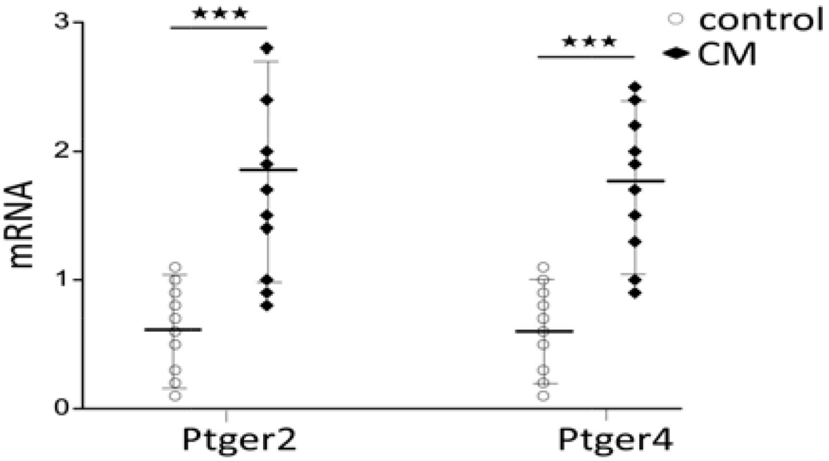

Supplementary Figure 3: The expression of EP2 and EP4 on CTLs cultured with cancer-infiltrating primary cell condition medium (CM). As shown in Supplementary Figure 3, Ptger2 and Ptger4 mRNA was measured using qRT-PCR. The expression of EP2 and EP4 was upregulated on CTLs cultured with CM from cancer ascites cells. Statistical analysis was performed by unpaired two-tailed *t*-test. $^{***}P < 0.001$.

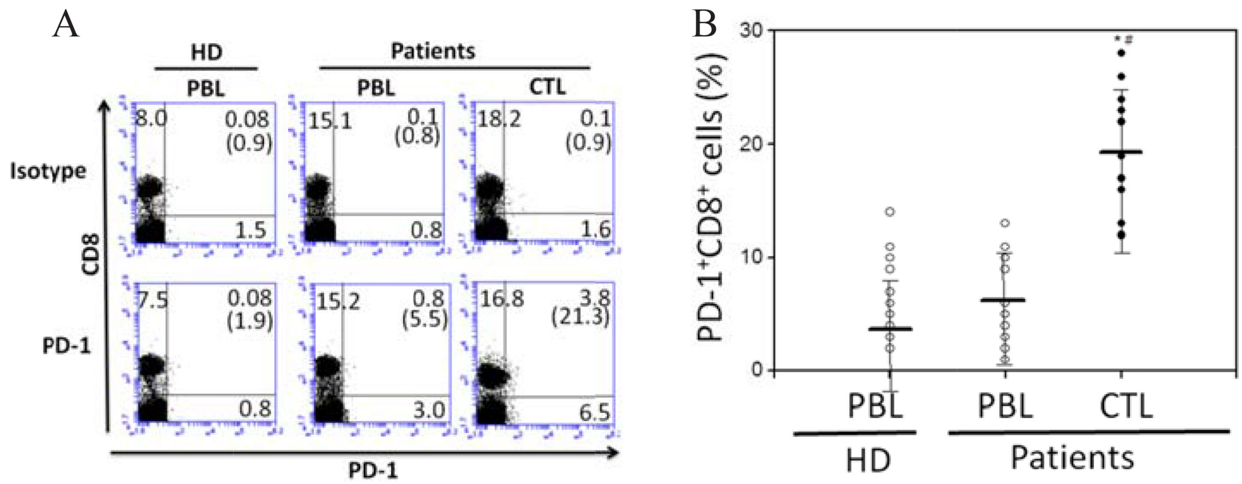

Supplementary Figure 4: PD-1^{+}CD8^{+} T cells preferentially accumulate at the tumor site. PD-1 expression is up-regulated at the tumor site of patients. (**A**) PBLs or CTLs were stained with anti-PD-1 or isotype-matched antibodies and analyzed by flow cytometry. Numbers on parentheses represent the percentage of PD-1^{+}CD8^{+} T cells. (**B**) Summary of the proportions of PD-1^{+}CD8^{+} cells in samples from several healthy donors (HD) and patients. *$P < 0.05$ compared with healthy donors' PBL. $^{\#}P < 0.05$ compared with patients' PBL.

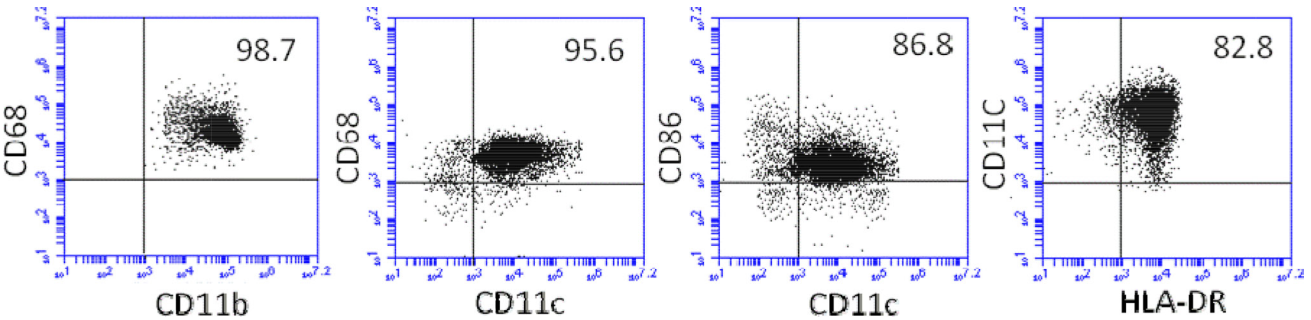

Supplementary Figure 5: Isolation APCs derived from tumors. CD11b myeloid cells were purified from tumor cell suspension using the MACS method (Miltenyi Biotec, Gladbach, Germany) according to the manufacturer's instructions. Briefly, cells were incubated with magnetic beads conjugated with anti-human CD11b and positively selected on LS columns. As shown in Supplementary Figure 5, The purity of recovered APCs assessed by flow cytometry was > 95%, which also co-express CD86 (> 85%) and MHC class II (> 80%).
